# *Pyropia Yezoensis* Extract Suppresses IFN-Gamma- and TNF-Alpha-Induced Proinflammatory Chemokine Production in HaCaT Cells via the Down-Regulation of NF-κB

**DOI:** 10.3390/nu12051238

**Published:** 2020-04-27

**Authors:** Yuna Ha, Won-Hwi Lee, JaeWoo Jeong, Mira Park, Ju-Young Ko, Oh Wook Kwon, Jongsung Lee, Youn-Jung Kim

**Affiliations:** 1 Research Institute of Basic Sciences, Incheon National University, Incheon 22012, Korea; dbsk335@hanmail.net (Y.H.); dnjsgnl_@naver.com (W.-H.L.); wjdowdn4@naver.com (J.J.); mira0295@inu.ac.kr (M.P.); herolegend@hanmail.net (J.-Y.K.); hades770@hanmail.net (O.W.K.); 2 Department of Cosmetic Science and Management, Graduate School, Incheon National University, Incheon 22012, Korea; 3 Department of Marine Sciences, Incheon National University, Incheon 22012, Korea; 4 Department of Genetic Engineering, College of Biotechnology and Bioengineering, Sungkyunkwan University, Suwon City 164-19, Gyunggi Do, Korea

**Keywords:** *Pyropia yezoensis*, AD, TARC, MDC, astaxanthin, xanthophyll, mitogen-activated protein kinases, NF-κB

## Abstract

*Pyropia yezoensis*, a red alga, is popular and harvested a lot in East Asia and is famous for its medicinal properties attributable to its bioactive compounds including amino acids (porphyra-334 and shinorine, etc.), polysaccharides, phytosterols, and pigments, but its anti-inflammatory effect and mechanism of anti-atopic dermatitis (AD) have not been elucidated. In this study, we investigate the anti-AD effect of *P. yezoensis* extract (PYE) on mRNA and protein levels of the pro-inflammatory chemokines, thymus, and activation-regulated chemokine (TARC/CCL17) and macrophage-derived chemokine (MDC/CCL22), in human HaCaT keratinocyte cells treated to interferon (IFN)-γ or tumor necrosis factor (TNF)-α (10 ng/mL each). The effect of the PYE on extracellular signal-regulated kinase (ERK) and other mitogen-activated protein kinases (MAPKs) was related to its suppression of TARC and MDC production by blocking NF-κB activation in HaCaT cells. Furthermore, astaxanthin and xanthophyll from *P. yezoensis* were identified as anti-AD candidate compounds. These results suggest that the PYE may improve AD and contained two carotenoids by regulating pro-inflammatory chemokines.

## 1. Introduction

Atopic dermatitis (AD) is a chronic inflammatory skin disease. It has a complex pathogenesis involving genetic, immunological, and environmental factors that lead to a dysfunctional skin barrier and dysregulation of the immune system [1]. Millions of people, including children, suffer from AD, so the development of effective and safe treatments is needed.

Several studies have demonstrated that cytokines such as interferon-γ (IFN-γ) and tumor necrosis factor-α (TNF-α) stimulate epidermal keratinocytes that lead to the activation of signaling pathways involved in pro-inflammatory responses [2,3]. Therefore, this model is usually used as an in vitro testing method for anti-inflammatory skin treatment agents. In addition, it has been shown that the expression of thymus and activation-regulated chemokine (TARC/CCL17) and macrophage-derived chemokine (MDC/CCL22) was significantly increased in keratinocytes of the lesion skin of AD patients [4,5]. Therefore, the downregulation of TARC and MDC production in keratinocytes might be an effective strategy for the treatment of inflammatory skin diseases such as AD [6,7]. 

The increased understanding of signal transduction mechanisms and gene regulation involved in the immune response has provided us with opportunities to discover novel natural resource therapeutic compounds that are useful for treating inflammatory disorders. One of the most studied signaling routes is the mitogen-activated protein kinase (MAPK) signaling pathway, which plays a crucial role in many aspects of immune-mediated inflammatory responses [8,9]. For this reason, we try to intensively investigate whether the MAPK-related signaling pathway could be used as a target for the treatment of human skin inflammation diseases such as AD.

*Pyropia yezoensis* (*P. yezoensis*) is a red alga mainly found in Korea, Japan, and China and is consumed as food. Edible marine algae contain a range of components with potential health benefits [10,11]. *P. yezoensis* is known to afford protective effects against UV and have antioxidant [12,13,14], anticancer [15], anti-inflammatory [16,17], antihypertensive [18], and tissue-healing properties [19,20,21]. It also contains numerous bioactive compounds, such as amino acids (porphyra-334 and shinorine, etc.) polysaccharides, phytosterols, and pigments (ß-carotene and astaxanthin, etc.) and is a promising candidate for useful cosmetic resources. 

Although *P. yezoensis* extract has been shown to have various biological activities, its effect as an anti-inflammatory agent for AD remains poorly understood. This study is designed to evaluate the anti-inflammatory effects of *P. yezoensis* extract on IFN-γ- and TNF-α-induced immune responses in an HaCaT human keratinocyte model.

## 2. Materials and Methods

### 2.1. Preparation of Pyropia Yezoensis Extracts (PYE), Astaxanthin (AS), and Xanthophyll (X)

The *P. yezoensis* was a kind gift from Professor Taejun Han (Ghent University Global Campus, Incheon, Korea). Astaxanthin and xanthophyll were purchased from Sigma-Aldrich (Sigma-Aldrich, St. Louis, MO, USA). For extract preparation, the *P. yezoensis* was washed with tap water to remove the salts and a dried sample (100 g) was pulverized, followed by extraction with 80% methanol (MeOH, 1:10, *w/v*) for 30 min by sonication. Then, it was filtered by Whatman filter paper and the solvents of filtrates were evaporated by the vacuum rotary evaporator. In this study, the water fraction of the *P. yezoensis* extract (PYE) was isolated as the supernatant and then lyophilized using a freeze-dryer (TD5508, Ilshin Lab, Co., Ltd., Yangju, Korea). The PYE, astaxanthin, and xanthophyll were dissolved in dimethyl sulfoxide (DMSO) before use in the experiments.

### 2.2. Cell Culture

The HaCaT cell line was purchased from American Type Culture Collection (ATCC, Manassas, VA, USA) and cultured in Dulbecco’s modified Eagle’s medium (DMEM; Gibco, Grand Island, NY, USA) supplemented with 10% fetal bovine serum (FBS; Gibco, Grand Island, NY, USA) and penicillin-streptomycin (100 U/mL; Gibco, Grand Island, NY, USA) at 37 °C exposed to 5% CO_2_ in a humidified incubator. The medium was changed every 3 days. HaCaT cells were seeded at a density of 1.5 × 10^5^ cells/dish in complete medium in a 100 mm cell culture dish. After 24 h, the medium was changed to serum-free DMEM containing the indicated concentrations of PYE (40, 200, and 1000 μg/mL) and 10 ng/mL of TNF-α (BD Biosciences, San Jose, CA, USA) or 10 ng/mL of IFN-γ (BD Biosciences, San Jose, CA, USA) were added to the cells. After 24 h, the cells were collected for western blot analysis and real-time RT-PCR. 

### 2.3. Cytotoxicity Assay

For the cytotoxicity assay, HaCaT cells were plated in a 96-well plate at a density of 1.5 × 10^4^ cells/dish for 24 h and treated with 10–2000 μg/mL PYE for 24 h. Then, the cells were incubated with 3-(4,5-dimethylthiazol-2yl)-2,5-diphenyl-2H-tetrazolium bromide (MTT; Sigma-Aldrich, St. Louis, MO, USA) (0.5 mg/mL) for 3 h. Following this, the medium was removed, and 100 μL DMSO was added to each well to dissolve the formazan crystals and the MTT metabolite. After thoroughly mixing the plate, the optical density was read at 560 nm, which directly correlates with the cell number.

### 2.4. RNA Extraction and cDNA Synthesis 

Total RNA was extracted using TRIzol reagent^TM^ (Invitrogen, Carlsbad, CA, USA), according to the manufacturer’s recommendations, and resuspended in diethyl pyrocarbonate (DEPC)-water. The quantity and purity of the RNA was verified at 260/280 nm using a NanoDrop® spectrophotometer (DaeMyung Science, Seoul, Korea). Purification was performed using an RNeasy mini kit (Qiagen, NY, USA). RNA samples were stored at −70 °C until used. The complementary DNA was synthesized from 500 ng total RNA using a Toyobo cDNA kit (Toyobo, Tokyo, Japan). 

### 2.5. Real-Time qPCR

The real-time PCR analysis was performed using an Applied CFX Connect^TM^ real-time PCR detection system (Bio-Rad Laboratories, Hercules, CA, USA). The PCR reaction mixture was prepared using SYBR Green Real-time PCR Master Mix (Toyobo, Osaka, Japan), according to the manufacturer's instructions. The system operates using a thermal cycler and a laser directed via fiber optics to each of the 96 sample wells. The thermal cycling conditions were 60 °C for 2 min and 95 °C for 10 min, followed by 40 cycles of 95 °C for 30 s, 60 °C for 30 s, and 72 °C for 30 s. The relative expression of the target genes was calculated using the CFX Connect^TM^ Real-Time PCR detection system employing the ΔΔCq method. TARC, MDC, and glyceraldehyde 3-phosphate dehydrogenase (GAPDH) oligonucleotide primers were purchased from Bioneer (Seoul, Korea). The primer sequences used are shown in Table 1.

### 2.6. Enzyme-Linked Immunosorbent Assay (ELISA) for MDC and TARC

The HaCaT cells (1.5 × 10^5^ cells/dish) were seeded in a 100 mm dish. After 24 h, the cells were incubated with IFN-γ or TNF-α (10 ng/mL each) in the presence or absence of PYE at the indicated concentrations for 24 h. The culture supernatants were collected 24 h after treatment and stored at −70 °C. They were analyzed for MDC and TARC using ELISA (R&D System, Minneapolis, MN, USA), according to the manufacturer’s instructions. The optical density was measured at 450 nm using a microplate reader (Varioskan Lux, Thermo Fisher Scientific, Waltham, MA, USA). 

### 2.7. Preparation of Whole Cell Lysates and Nuclear Extracts

Whole cell lysates and nuclear extracts were separated as described previously [22]. HaCaT cells were incubated with IFN-γ or TNF-α in the presence or absence of PYE, astaxanthin and xanthophyll for 24 h, and the whole cell lysates and nuclear extracts were separated using a nuclear extract kit (Active Motif, Carlsbad, CA, USA). Briefly, cells (1.5 × 10^5^ cells/dish) were washed with cold PBS containing phosphatase inhibitors and collected. To obtain whole cell lysates, cells were lysed in 300 μL complete lysis buffer, mixed completely, and centrifuged at 14,000 × *g* for 20 min at 4 °C. Supernatants were then stored at −80 °C until use. For nuclear extracts, after washing the cells, they were lysed in hypotonic buffer and centrifuged at 14,000 × *g* for 30 s at 4 °C as previously described. After separating the supernatant, the pellets were resuspended in 50 μL of complete lysis buffer and centrifuged at 14,000 × *g* for 10 min at 4 °C, and the supernatants were stored at −80 °C until use.

### 2.8. Western Blot

The protein concentration was determined using the bicinchoninic acid (BCA) protein assay, and equal amounts of protein in the whole cell lysates were separated using sodium dodecyl sulfate (SDS)-polyacrylamide gel electrophoresis (PAGE). Then, total protein was transferred to the polyvinylidene fluoride (PVDF) membrane (Bio-Rad Laboratories, Hercules, CA, USA) prior to blocking using 5% skim milk or 2–5% bovine serum albumin (BSA) in Tris-buffered saline-Tween 20 (TBS-T) solution [23]. The primary antibodies for extracellular signal-regulated kinase (ERK1/2), phospho-ERK1/2, c-Jun N-terminal kinase (JNK1/2), phospho-JNK1/2, p38 MAPK, phospho-p38 MAPK, IκBα, phospho-NF-κB, histone h1, α-tubulin, and GAPDH (Cell Signaling Technology Inc., Beverly, MA, USA) were incubated with the PVDF membrane overnight at 4 °C. Then, the secondary antibody (1:2000 in 5% BSA in TBS-T solution) was incubated for 1 h at room temperature (Cell Signaling Technology Inc., Beverly, MA, USA). All primary antibodies were diluted 1:1000 with 5% skim milk or 2–5% BSA in TBS-T solution. For protein detection, antibodies reacted with conjugated to horseradish peroxidase (HRP) and detected using enhanced chemiluminescence (ECL) to the secondary antibody for 1 min at room temperature. Target protein size were detected and photographed using Chemidoc XRS (Bio-Rad Laboratories, Hercules, CA, USA).

### 2.9. High Performance Liquid Chromatography (HPLC) Analysis

Chromatographic analyses were performed on an Agilent HPLC system, 1200 series (Agilent Technologies, Inc. Waldbronn, Germany) equipped with double-beam photometer (Variable Wavelength Dector). UV absorbance was monitored at 470 nm. Quantification was analyzed by integration of the peak areas at 470 nm. The separation was performed using a Kromasil 100-5C_18_ column (particle size, 5 μm; 250 * 4.6 mm; Teknokroma, Barcelona, Spain) with the temperature maintained at 30 °C, and the injection volume was 10 μL. The mobile phase consisted of A: 0.05% Trifluoracetic Acid H_2_O and B: acetonitrile, and the flow rate was 1.0 mL/min. A gradient of 0–20 min from 10–0% and 20–30 min at 100% B was used. The PYE and astaxanthin (Sigma-Aldrich, St. Louis, MO, USA) were used as the sample and standard at a concentration of 10 mg/mL and 0.1 μg/mL of each of astaxanthin and xanthophyll (Sigma-Aldrich, St. Louis, MO, USA) was used for HPLC analysis to identify the peaks.

### 2.10. Statistical Analysis

The data analysis used the GraphPad Prism version 5.0 (GraphPad Software, San Diego, CA, USA). The results are expressed as the mean ± standard error of the mean (S.E.M) or standard deviation (SD) of triplicate experiments. Statistical comparisons between of different treatments were performed using one-way ANOVAs with Tukey’s multiple comparison post-hoc tests. * *p* < 0.05, ** *p* < 0.01, and *** *p* < 0.001 were considered to be statistically significant.

## 3. Results

### 3.1. Effect of P. Yezoensis Extract (PYE) on the Viabillity of HaCaT Cells

**Figure 1:**
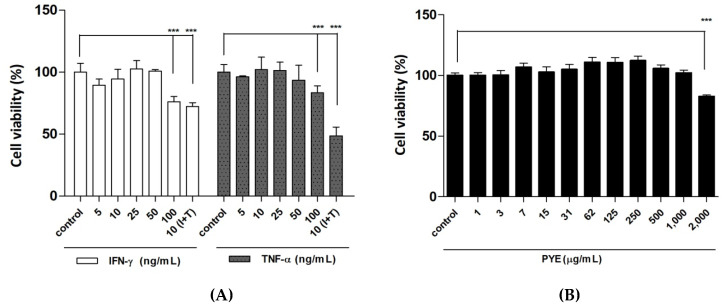
Cell viability at various treatment concentrations of interferon (IFN)-γ, tumor necrosis factor (TNF)-α, and *Pyropia yezoensis* extract (PYE) on HaCaT cells. Cells were incubated with the indicated concentration of each cytokine (IFN-γ (I) and TNF-α (T)), alone or (I+T) combined for 24 h (**A**). HaCaT cells were treated with various treatment concentrations of PYE for 24 h (**B**). Cell viability was then determined by the 3-(4,5-dimethylthiazol-2yl)-2,5-diphenyl-2H-tetrazolium bromide (MTT) assay. Results are expressed as means ± standard deviation (SD) of triplicate experiments. Statistical comparisons of different treatments were performed using one-way analysis of variance (ANOVA) with Tukey’s multiple comparison test. Note: *** *p* < 0.001 compared with the control group.

To determine the effects of cytokine (IFN-γ or TNF-α) stimulation and varying concentrations of PYE on the HaCaT cell viability, the MTT assay was performed. As shown in Figure 1A, 24-h treatment with the cytokines (IFN-γ and TNF-α each) at concentrations ranging from 5 to 10 ng/mL exerted no significant cytotoxicity on HaCaT cells. In contrast, treatment with the IFN-γ combined TNF-α induced a significant cytotoxicity on these cells at 10 ng/mL. In addition, PYE did not exert a significant cytotoxicity on these cells, except at 2000 μg/mL (Figure 1B). Therefore, we used each cytokine (IFN-γ or TNF-α) at 10 ng/mL and the PYE at 40, 200, and 1000 μg/mL in subsequent experiments.

### 3.2. PYE Suppressed IFN-γ and TNF-α induced mRNA Expression Level of TARC and MDC in HaCaT Cells

**Figure 2:**
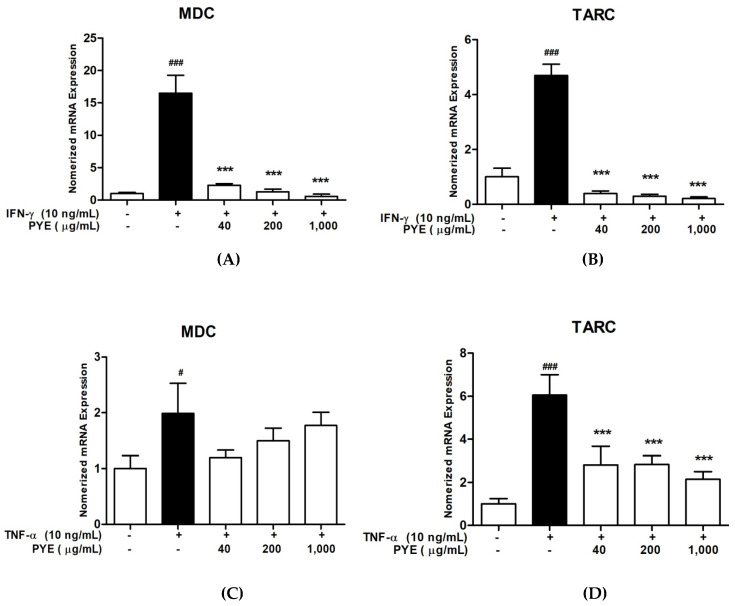
Effects of PYE on mRNA expression of macrophage-derived chemokine (MDC) and thymus, and activation-regulated chemokine (TARC) in HaCaT cells stimulated with IFN-γ (**A**,**B**) or TNF-α (**C**,**D**). HaCaT cells were co-treated with PYE and IFN-γ or TNF-α (10 ng/mL) for 24 h. After treatment, the mRNA expression of MDC and TARC was measured and normalized to glyceraldehyde 3-phosphate dehydrogenase (GAPDH) expression. Values are means ± standard error of mean (SEM) of three independent experiments. Statistical comparisons of different treatments were performed using one-way analysis of variance (ANOVA) with Tukey’s multiple comparison test. Note: ### *p* < 0.001 and # *p* < 0.01 control group vs. the IFN-γ or TNF-α (10 ng/mL)-treated group, and *** *p* < 0.001 IFN-γ or TNF-α (10 ng/mL)-treated group vs. PYE co-treated group.

To investigate the effect of PYE on pro-inflammatory chemokine (TARC and MDC) production by TNF-α/IFN-γ-stimulation, HaCaT cells were treated with PYE and IFN-γ or TNF-α for 24 h, and the mRNA expression of MDC and TARC chemokines was analyzed using real-time qPCR. As shown in Figure 2A,B, PYE (40 to 1000 μg/mL) significantly inhibited the mRNA expression of IFN-γ-stimulated TARC and MDC in a dose-dependent manner. Similarly, PYE suppressed the TNF-α-stimulated mRNA expression of TARC, but weakly inhibited MDC (Figure 2C,D). These results suggest that PYE can modulate the mRNA expression of the pro-inflammatory chemokines TARC and MDC.

### 3.3. PYE Decreased the IFN-γ- and TNF-α-induced Production of TARC and MDC in HaCaT Cells

**Figure 3:**
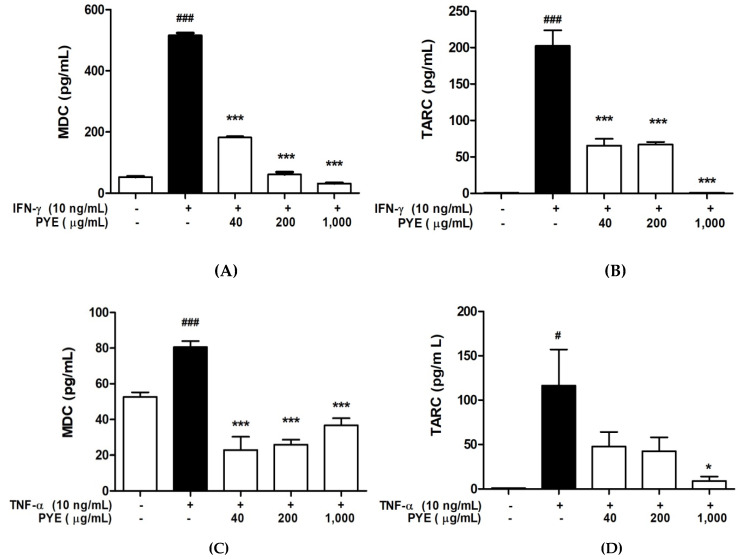
Effects of PYE on the TNF-α/IFN-γ-induced production of chemokines in HaCaT cells. The cells were treated with IFN-γ (**A**,**B**) or TNF-α (**C**,**D**) (10 ng/mL) in the absence or presence of varying concentrations of PYE for 24 h. After treatment, cell supernatants were measured by ELISA. Data are presented as means ± standard deviation (SD) of triplicate experiments conducted in duplicate. Note: ### *p* < 0.001 and # *p* < 0.01 control group vs. the IFN-γ or TNF-α (10 ng/mL)-treated group, and * *p* < 0.05 and *** *p* < 0.001 IFN-γ or TNF-α (10 ng/mL)-treated group vs. PYE co-treated group.

We investigated the effect of PYE on the production of TARC and MDC chemokines induced by IFN-γ or TNF-α. HaCaT cells were incubated with the medium alone or IFN-γ or TNF-α in the presence or absence of PYE at indicated concentrations. The levels of TARC and MDC in the culture medium supernatant were determined by ELISA (Figure 3). Even at 10 ng/mL, IFN-γ increased the secretion of TARC from a basal level of 1 pg/mL to 202.43 ± 36.78 pg/mL (*p* < 0.001) and MDC from a basal level of 52.64 ± 4.47 to 516.1 ± 13.19 pg/mL (*p* < 0.01). TNF-α stimulation increased the secretion of TARC from a basal level of 1 pg/mL to 116.41 ± 70.56 pg/mL (*p* < 0.05). Additionally, as a result of mRNA expression, the secretion of these chemokines in HaCaT cells was significantly inhibited by PYE compared to the IFN-γ- or TNF-α-stimulated group (*p* < 0.001). The results showed that PYE inhibited the release of IFN-γ- and TNF-α-stimulated pro-inflammatory chemokines TARC and MDC. 

### 3.4. Effect of PYE on the Phosphorylation of MAPKs in IFN-γ- and TNF-α-induced HaCaT Cells

**Figure 4:**
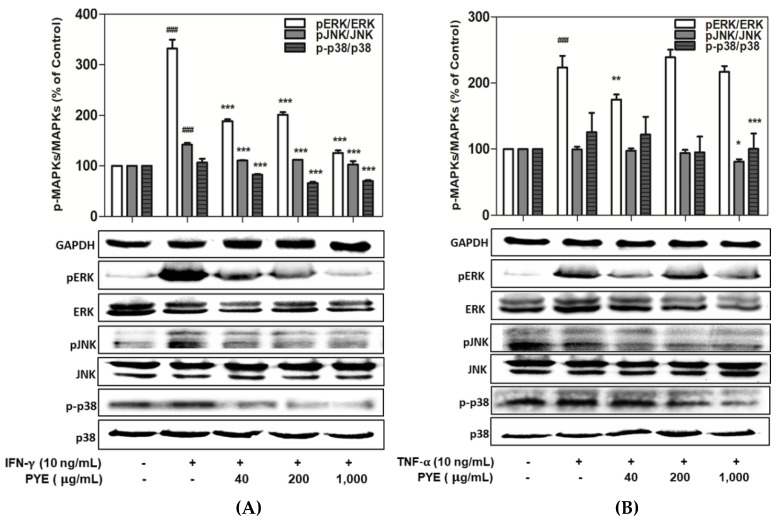
Effect of PYE on the mitogen-activated protein kinase (MAPK) signaling pathway in HaCaT cells. Protein was isolated from cells treated with IFN-γ (**A**) or TNF-α (**B**) in the absence or presence of varying concentrations of PYE for 24 h. Phosphorylation levels of extracellular signal-related kinase (ERK), c-Jun N-terminal kinase (JNK), and p38 determined using Western blot analysis. Equal amounts of protein loading were checked using total protein and housekeeping protein GAPDH antibodies. Relative levels of p-ERK, p-JNK, and p-p38 were calculated using Image Lab. Values are means ± standard deviation (SD) of independent experiments. Note: ### *p* < 0.001 control group vs. the IFN-γ or TNF-α (10 ng/mL)-treated group, and * *p* < 0.05, ** *p* < 0.01, and *** *p* < 0.001 IFN-γ or TNF-α (10 ng/mL)-treated group vs. PYE co-treated group.

To investigate the molecular mechanism of the anti-inflammatory effect of PYE in IFN-γ- or TNF-α-stimulated HaCaT cells, we examined whether PYE inhibits the activation of MAPKs. HaCaT cells were treated with PYE (40, 200, and 1000 µg/mL) and IFN-γ or TNF-α for 24 h and cell lysates were collected for an analysis of the activities of MAPKs, such as ERK, JNK, and p38, by Western blotting. As shown in Figure 4A, various concentrations of PYE (40–1000 μg/mL) strongly inhibited the phosphorylation of ERK, JNK, and p38 in the IFN-γ-stimulated HaCaT cells. In comparison, HaCaT cells stimulated by TNF-α only activated ERK significantly, but JNK and P38 were not activated. In addition, no significant inhibition was observed after PYE treatment. The enhanced ERK, JNK, and p38 expression induced by TNF-α was downregulated by PYE or no treatment (Figure 4B). These results demonstrated the regulatory effect of PYE on the activation of MAPKs in HaCaT cells, as determined using Western blot analysis.

### 3.5. Effect of PYE on NF-κB Translocation in IFN-γ- and TNF-α-induced HaCaT Cells

**Figure 5:**
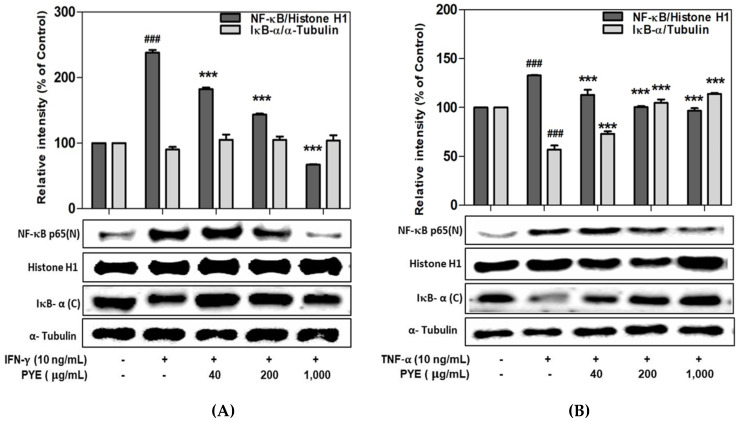
Effect of PYE on the translocation of nuclear factor NF-kB/IkB-α induced by IFN-γ (**A**) or TNF-α (**B**) in HaCaT cells. HaCaT cells were incubated with various concentrations of PYE (40–1000 μg/mL) and stimulated with IFN-γ or TNF-α (10 ng/mL). IκB-α (**C**) in cytosol extract; NF-κB (N) in nuclear extract. Expression of NF-κB and IκB-α was determined using Western blotting. Densitometry analysis presented as the relative intensity of housekeeping proteins was used as a loading control. Data are means ± standard deviation (SD) of independent experiments. Note: ### *p* < 0.001 control group vs. the IFN-γ or TNF-α (10 ng/mL)-treated group, and *** *p* < 0.001 IFN-γ or TNF-α (10 ng/mL)-treated group vs. PYE co-treated group.

NF-κB is a transcription factor that is critically involved in AD-related signaling in IFN-γ- or TNF-α-induced HaCaT cells. Therefore, the regulation of NF-κB expression is important for the management of skin disease. As shown in Figure 5A, PYE suppressed IFN-γ-induced NF-κB activation. Additionally, the TNF-α-induced nuclear translocation of NF-κB was significantly inhibited by PYE, whereas IκB-α was increased by degradation (Figure 5B). Therefore, these results postulate that PYE may inhibit the expression of proinflammatory cytokine via the down-regulation of NF-κB activation.

### 3.6. Analysis of Candidate Carotenoid Substances in PYE by HPLC

**Figure 6:**
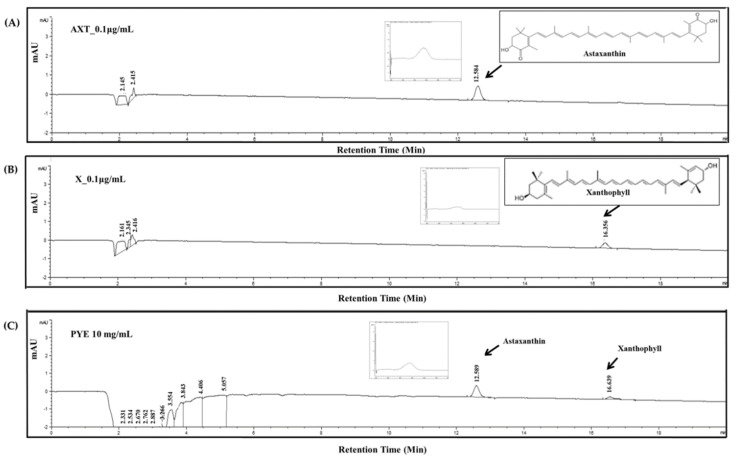
The representative chromatograms of (**A**) astaxanthin (AXT) standard at 0.1 μg/mL, (**B**) xanthophyll (X) standard at 0.1 μg/mL, and (**C**) *Pyropia yezoensis* extract (PYE) at 10 mg/mL detected by high-performance liquid chromatography (HPLC) with a diode-array detector (DAD) at 470 nm. The flow rate was 1.0 mL/min, and the sample injection volume was 50 μL.

We analyzed prior studies and the component analysis to identify which components exhibit anti-inflammatory activity. As a primary study, the total flavonoid, polyphenol, and carotenoid content was measured and compared to other seaweeds (Supplementary Table S1). We found that the total carotenoid content of PYE is higher than that of other seaweeds. As a result of HPLC analysis, two carotenoid components were detected in PYE. The chromatogram of PYE is shown in Figure 6C. The two peaks observed were assigned to astaxanthin and xanthophyll carotenoid by comparing their retention time with a standard compound of astaxanthin and xanthophyll in the chromatogram at 470 nm. There are 0.08 μg/mL of astaxanthin and 0.05 μg/mL xanthophyll in 10 mg/mL of the PYE extract. The present results contribute to the elucidation of the anti-AD effect of astaxanthin and xanthophyll, which are carotenoids in red algae.

### 3.7. Effect of Astaxanthin and Xanthophyll on the NF-kB/IkB-α and MAPK Signaling Pathway in IFN-γ- or TNF-α-Treated HaCaT Cells

**Figure 7:**
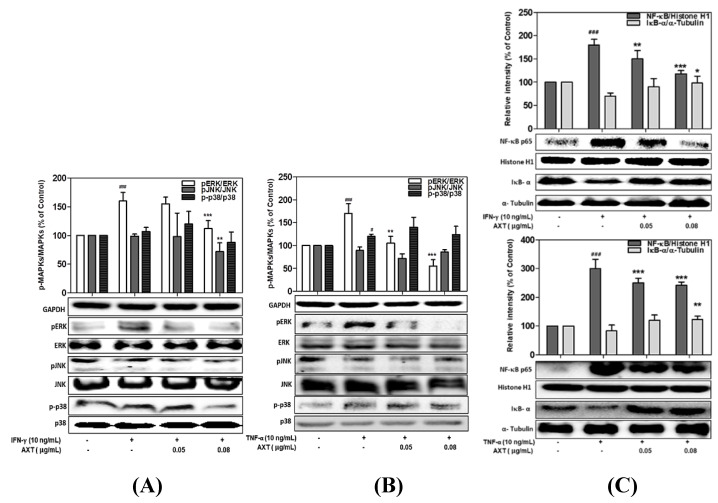

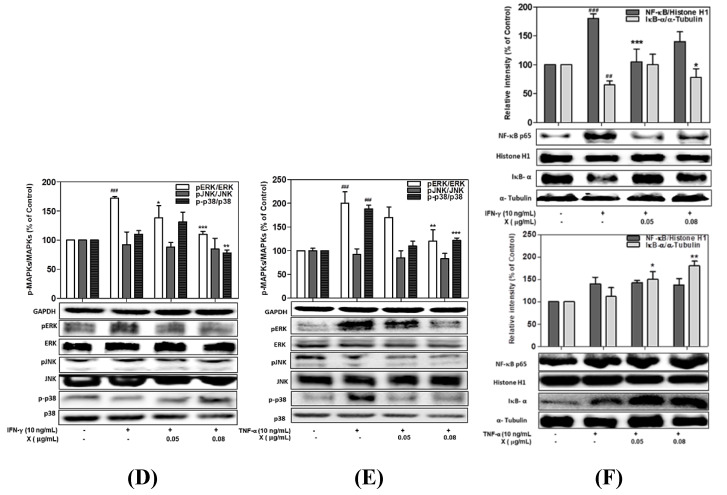
Effects of astaxanthin (AXT) and xanthophyll (X) on the MAPKs and NF-κB/IκB-α signaling pathway in IFN-γ or TNF-α-treated HaCaT cells. HaCaT cells were incubated with two concentrations of AXT and X (0.05–0.08 μg/mL) and stimulated with IFN-γ or TNF-α (10 ng/mL). Quantitative band-intensity for p-MAPKs (**A**,**B**,**D**,**E**) and p- NF-κB/IκB-α (**C**,**F**) were analyzed by densitometry, normalized to the level of GAPDH, histone H1, or α-tubulin, and calculated as a percentage of the basal response. Data are means ± standard deviation (SD) of independent experiments. Note: Significant differences # *p* < 0.05, ## *p* < 0.01, and ### *p* < 0.001 from control vs. the TNF-α/IFN-γ-(10 ng/mL)-induced group, and * *p* < 0.05, ** *p* < 0.01, and *** *p* < 0.001 IFN-γ or TNF-α (10 ng/mL)-induced group vs. astaxanthin and xanthophyll treated group.

It was investigated whether the astaxanthin (AXT) and xanthophyll (X) contained in PYE influence the NF-κB / IκB-α and MAPK signaling pathways in mechanisms similar to PYE.As shown in Figure 7A, ERK, JNK, and p38 phosphorylation ratio in IFN-γ-induced HaCaT cells was inhibited by astaxanthin up to 123.7%, 82.5%, and 87.8% (on 0.08 μg/mL concentration), respectively. Furthermore, astaxanthin (0.08 μg/mL) strongly inhibited the phosphorylation of ERK and JNK in the TNF-α-stimulated (Figure 7B). Additionally, similar to PYE results, NF-κB translocation significantly inhibited by astaxanthin on IFN-r and TNF-a stimulated in HaCaT cells, respectively (Figure 7C).

In the xanthophyll-treated group, the ratio of ERK and p38 phosphorylation in IFN-γ-stimulated HaCaT cells increased to 172.3% and 110.3%, respectively, compared to the control group. However, after treatment with xanthophyll (0.08 μg/mL), the p-ERK and p-p38 were reduced 110.2% and 78.1%, respectively (Figure 7D). In addition, in the case of cells stimulated with TNF-α, it was confirmed that p-ERK decreased 202% to 120.7% when treated with xanthophyll, and p-p38 decreased significantly from 188% to 122.2% (Figure 7E). Finally, it was confirmed that the NF-κB translocation of cells stimulated with IFN-γ and TNF-α was reduced to 139.4% and 138.3% after treatment with xanthophyll (0.08 μg/mL) (Figure 7 F). More importantly, astaxanthin and xanthophyll reduced MAPK and NF-κB signal activation, and these results showed similar results to that of PYE. These results suggest that astaxanthin and xanthophyll play a key role in the anti-inflammatory effect of PYE.

## 4. Discussion

In Asia, *Pyropia yezoensis* (*P. yezoensis*) is used as a traditional medicine in the treatment of various diseases. *P. yezoensis* has numerous biological functions, including anti-inflammation, anti-oxidant, anti-cancer, anti-photo aging, and anti-hypertensive effects [13,24,25,26]. Recently, many studies examined the biological effects of *P. yezoensis*, but studies on atopic dermatitis (AD) from *P. yezoensis* extract (PYE) have not been reported until now. In this study, we revealed that PYE treatment reduced the production of inflammatory-mediated chemokines in IFN-γ/TNF-α-induced HaCaT cells through the inactivation of NF-κB and MAPK pathway.

Keratinocytes produce pro-inflammatory cytokines and chemokines, which are affected in the development of inflammatory skin disorders, such as AD. AD is a Th2-type skin disease characterized by inflammation, in which lymphocytes infiltrate into the dermis. TARC and MDC are Th2 chemokines that bind to and attract CCR4+ Th2 cells to sites of inflammatory tissue [27,28,29]. Previous studies have evidenced that TARC is highly expressed in the basal epidermis of lesional skin in NC/Nga mice and AD patients. Additionally, a high level of MDC was observed in the monocyte-derived dendritic cells isolated from venous blood in patients with AD and lesional skin of AD [30,31]. Therefore, TARC and MDC are thought to play important roles in the pathogenesis of AD. We performed experiments to explore the effect of PYE on the IFN-γ/TNF-α-induced expression of TARC and MDC in HaCaT cells. PYE significantly inhibited the protein and mRNA expression of TARC and MDC in IFN-γ/TNF-α-induced HaCaT cells (Figure 2 and Figure 3). 

In keratinocytes, IFN-γ/TNF-α activates several intracellular signaling pathways, including those of mitogen-activated protein kinases (MAPKs) [32]. The MAPK cascade plays an important role in immune responses, regulates multiple cellular processes, including gene expression, cell death, and cell proliferation and is involved in the production of inflammatory chemokines [33,34]. The suppression of MAPKs has been reported to reduce the synthesis of pro-inflammatory cytokines and their intracellular signaling pathways and inhibit the activation of NF-κB [35,36]. IFN-γ/TNF-α-induced HaCaT cells were confirmed to activate MAPKs, including ERK or JNK, and p38 (Figure 4A,B). IFN-γ is known to activate the ERK and JNK pathways in murine macrophages and the p38 MAPK pathway in keratinocytes [37]. Additionally, TNF-α- stimulates the activation of various signaling molecules, including ERK, JNK, and p38 MAPK, in many cell types [38]. We investigated the inhibitory effect of PYE in MAPK activation on IFN-γ/TNF-α-induced keratinocytes. These results showed that PYE reduces the production of pro-inflammatory cytokines and chemokines in HaCaT cells by inhibiting the phosphorylation of ERK, JNK, and p38 in a dose-dependent manner (Figure 5 A). Similar to our study, several natural compounds from marine algae have been shown to inhibit the actions of inflammatory chemokines by regulating MAPK cascades [39,40,41].

Previous reports have shown that the NF-κB signaling pathway regulates the production of TARC and MDC in HaCaT cells [42,43]. In this study, we showed that PYE inhibited the activation of the NF-κB signaling pathway. NF-κB is a major transcription factor that regulates the transcription of many genes related to the development of acute and chronic inflammatory diseases [44]. In steady states, NF-κB/p65 binds with IκB-α in the cytoplasm. Cytokines such as IFN-γ and TNF-α induce phosphorylation and degradation of IκB-α, leading to phosphorylation and nuclear translocation of NF-κB/p65. NF-κB then induces the expression of inflammatory related genes in nucleus [45]. PYE suppressed the activation of NF-κB and degradation of IκB-α induced by IFN-γ/TNF-α (Figure 4A,B). These results indicate that PYE leads to the inhibition of the production of TARC and MDC by inhibiting the NF-κB pathway in HaCaT cells. Previous studies have reported the inhibitory effect of natural products and extracts from marine algae against inflammatory skin disorders by targeting the signaling pathway, leading to NF-κB and STAT1 activation [46,47,48,49]. Eom et al. reported that *Elsenia bicyclis* inhibited pro-inflammatory cytokines and chemokines production in HaCaT cells by inhibiting NF-κB activation [46]. Cho et al. recently reported that the eckol compound from *Ecklonia cava* suppressed MDC and TARC production by blocking MAPKs and NF-κB signaling pathways in HaCaT cells [47]. 

In a previous study, we first measured the total carotenoid, polyphenol, and flavonoid content in PYE to identify substances with anti-inflammatory effects and compared them to other red-macro algae (Supplementary Table S1). We found that the total content of total carotenoids in PYE was the highest when compared to other extracts, which is why we focused on carotenoids when analyzing PYE components.

According to previous literature, we investigated the intermediate carotenoids of *P. yezoensis* and found that carotenoids include both α-carotene, β-carotene. The α-cryptoxanthin and zeinoxanthin, which are monohydroxy-carotenoids, are produced as intermediates when lutein synthesis in α-carotene of *P. yezoensis*. Carotenoid compounds contribute to light harvesting and photo-protection and have potent antioxidant, anti-inflammatory, and neuroprotective properties [50]. Astaxanthin has been reported to have anti-inflammatory effects with nerve inflammation, gastric inflammation, and heat stress-induced inflammation [51,52,53]. The main sources of astaxanthin are known mainly isolated from natural krill shrimp, lobster, crab, salmon, yeast strains, and marine algae which are abundant in *Phaffia rhodozyma* and *Haematococcus pluvialis*. In many previous studies, xanthophyll is known to exist in *P. yezoensis.* However, it has been reported that their content might be different depending on environmental factors such as an inhabited area and temperature stress [54]. Furthermore, xanthophyll has been reported to have anti-inflammatory effects on brain inflammation and in LPS-stimulated inflammation [55,56].

Here, we confirm that astaxanthin and xanthophyll were detected in PYE among carotenoid components through HPLC analysis. Additionally, there was 0.08 μg/mL astaxanthin and 0.05 μg/mL xanthophyll in 10 mg/mL PYE. On the basis of these results, we observed the same signal transduction pathways compared to PYE and investigated the bioactive roles of astaxanthin and xanthophylls contained in PYE. We demonstrated that MAPK and NF-κB/ IκB-α signaling pathways in stimulated HaCaT cells treated with individual astaxanthin and xanthophylls similarly inhibited PYE. Therefore, we strongly expect that, due to the results, astaxanthin and xanthophyll are the main components that have anti-inflammatory effects on PYE.

Although our results suggest that the anti-AD effect of PYE on HaCaT cells may be attributed to astaxanthin and xanthophyll, other compounds present in PYE may also have an effect on the overall ability to inhibit pro-inflammatory chemokines. Through further study, it is expected that researchers will be able to identify more complex derivatives of carotenoids with inflammatory effects. Even though this analyzed data is preliminary, it is crucial because there has been no report on *P. yezoensis* extract regarding its effect on anti-AD.

**Figure 8:**
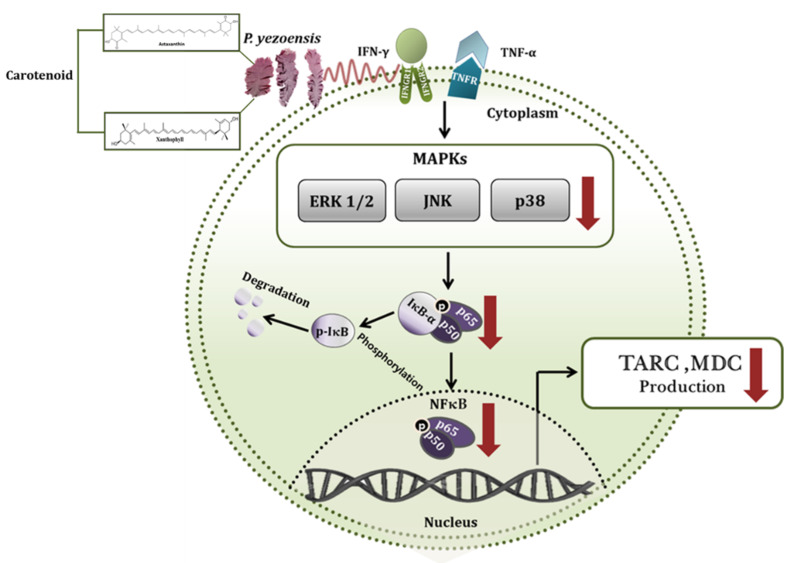
Schematic inhibitory signaling pathway of *P. yezoensis* extract (PYE) on interferon (IFN)-γ- and tumor necrosis factor (TNF)-α-induced TARC and MDC production in human keratinocytes. Red poly line arrows indicate the activity of *P. yezoensis* extract (PYE) and black arrows indicate the induction of cytokines.

In conclusion, this study investigated the basic mechanism of anti-inflammatory activity of PYE in HaCaT cells, as an in vitro model of human keratinocytes in inflammatory skin disease. In IFN-γ/TNF-α-induced HaCaT cells, PYE reduced the production of Th2 chemokines, such as TARC and MDC. The mechanism underlying these effects included the suppression of the phosphorylation of ERK, JNK, and p38 expression, and then the nuclear translocation of NF-κB/p65, by blocking IκB-α degradation (Figure 8). Moreover, we identified two carotenoid compounds and the results were similar to those of PYE. Therefore, we strongly expect that astaxanthin and xanthophyll are the main substances with anti-inflammatory effects in PYE. These results suggest the potential of PYE as a preventive agent in the treatment of AD. However, additional studies are needed to verify the anti-inflammatory activity of PYE in skin diseases using an AD animal model. 

## Figures and Tables

**Table 1:** Sequences of real-time reverse transcription polymerase chain reaction (RT-PCR) primers.

Genes	Sequences (5′→3′)
*GAPDH* (Forward)	CGT CTC CTC TGA CTT CAA CA
*GAPDH* (Reverse)	AGC CAA ATT CGT TGT CAT AC
*MDC* (Forward)	CAG CAC GAG GGA CCA ATG TG
*MDC* (Reverse)	CTT GGG GTC CGA ACA GAT GG
*TARC* (Forward)	ACT GCA CTC CTG GTT GTC CT
*TARC* (Reverse)	AAG GTT AGC AAC ACC ACG CC

GAPDH, glyceraldehyde 3-phosphate dehydrogenase; MDC, macrophage-derived chemokine; TARC, thymus and activation-regulated chemokine.

## Supplementary Materials

The following are available online at https://www.mdpi.com/2072-6643/12/5/1238/s1, Table S1: Total polyphenol, flavonoids, and carotenoid contents from seaweed extracts including *Pyropia yezoensis*.
