# Supplementary material for: Pyropia Yezoensis Extract Suppresses IFN-Gamma- and TNF-Alpha-Induced Proinflammatory Chemokine Production in HaCaT Cells via the Down-Regulation of NF-κB
Source: Nutrients. 2020 Apr 27;12(5):1238. doi: 10.3390/nu12051238 (PMC7285056; doi:10.3390/nu12051238)
Supplement: Supplementary file 1 [file nutrients-12-01238-s001.pdf]

Supplementary Table S1. Total polyphenol, flavonoids, and carotenoid contents from seaweed extracts including *Pyropia yezoensis*

Species	Total polyphenol content (g GAE/100g)	Total flavonoids content (g QE/100g)	Total Carotenoid content (g BE/1000g)
Pyropia yezoensis (80% MeOH)	6.27 ± 0.73	2.53 ± 0.18	3.55 ± 2.10
Polyopes affinis (80% EtOH)	0.55 ± 0.46	1.11 ± 0.17	1.38 ± 1.62
Polyopes affinis (70% MeOH)	4.37 ± 0.54	2.26 ± 0.54	0.88 ± 1.2
Gloiopeltis tenax (80% EtOH)	1.23 ± 0.36	2.84 ± 0.17	3.88 ± 1.6

Determination of total phenolics content (TPC), total flavonoids content (TFC) and beta carotene content (β-carotene) calibration curve were developed as galic acid (absorbance read at725 nm), quercetin (absorbance was read at 415 nm), and βcarotene (wavelength of 450 nm). GAE: gallic acid equivalent, QE: quercetin equivalent, BE: βcarotene equivalent.
